# Spike-Timing Theory of Working Memory

**DOI:** 10.1371/journal.pcbi.1000879

**Published:** 2010-08-19

**Authors:** Botond Szatmáry, Eugene M. Izhikevich

**Affiliations:** The Neurosciences Institute, San Diego, California, United States of America; Gatsby Computational Neuroscience Unit, University College London, United Kingdom

## Abstract

Working memory (WM) is the part of the brain's memory system that provides temporary storage and manipulation of information necessary for cognition. Although WM has limited capacity at any given time, it has vast memory content in the sense that it acts on the brain's nearly infinite repertoire of lifetime long-term memories. Using simulations, we show that large memory content and WM functionality emerge spontaneously if we take the spike-timing nature of neuronal processing into account. Here, memories are represented by extensively overlapping groups of neurons that exhibit stereotypical time-locked spatiotemporal spike-timing patterns, called *polychronous* patterns; and synapses forming such *polychronous* neuronal groups (PNGs) are subject to associative synaptic plasticity in the form of both long-term and short-term spike-timing dependent plasticity. While long-term potentiation is essential in PNG formation, we show how short-term plasticity can temporarily strengthen the synapses of selected PNGs and lead to an increase in the spontaneous reactivation rate of these PNGs. This increased reactivation rate, consistent with *in vivo* recordings during WM tasks, results in high interspike interval variability and irregular, yet systematically changing, elevated firing rate profiles within the neurons of the selected PNGs. Additionally, our theory explains the relationship between such slowly changing firing rates and precisely timed spikes, and it reveals a novel relationship between WM and the perception of time on the order of seconds.

## Author Summary

Working memory (WM) is the part of the brain's vast memory system that provides temporary storage and manipulation of the information necessary for complex cognitive tasks, such as language comprehension, learning, and reasoning. Despite extensive neuroscience research, its mechanism is not clearly understood. We exploit a well-known feature of the brain — its ability to use precisely timed spiking events in its operation — to show how working memory functionality can emerge in the brain's vast memory repertoire. Our neural simulations explain many features of neural activity observed *in vivo* during working memory tasks, previously thought to be unrelated, and our results point to a relationship between working memory and other mental functions such as perception of time. This work contributes to our understanding of these brain functions and, by giving testable predictions, has the potential to impact the broader neuroscience research field.

## Introduction

Various mechanisms have been proposed to model the main aspect of neural activity — elevated firing rates of a cue-specific population of neurons — observed during the delay period of a working memory (WM) task [1]–[4]. These include reentrant spiking activity [5], intrinsic membrane currents [6], NMDA currents [7]–[10], and short-term synaptic plasticity [7], [11], [12]. These mechanisms, however, fail to explain other aspects of neural correlates of WM [13], and they have been demonstrated to work only with a limited memory content where the number of items represented in long-term memory is small, i.e., they hold in WM a few items (limited capacity [14]) out of only a conceivable few (limited memory content). Memories in these simulated networks are often represented by carefully selected, largely non-overlapping groups [15] of spiking neurons [11]. Indeed, extending the memory content in such networks increases the overlap between the memory representations (unless the size of the network is increased, too), and activation of one representation spreads to others, resulting in uncontrollable epileptic-like “runaway excitation”. The narrow memory content is at odds with experimental findings that neurons participate in many different neural circuits (see e.g. [16]–[18]) and, therefore, are part of many distinct representations that form a vast memory content for WM. These limitations may be overcome by a model that accounts for the precise spike-timing nature of neural processing.

**Figure 1:**
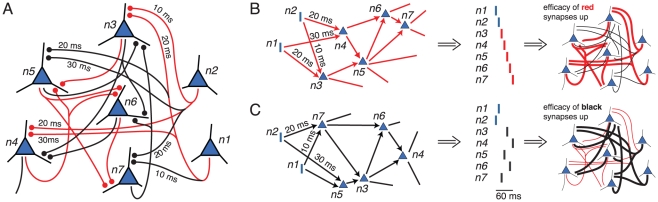
Illustration of *polychronous* neuronal groups and associative short-term plasticity. (A) Synaptic connections between neurons *n*1, *n*2, …, *n*7 have different axonal conduction delays arranged such that the network forms two functional subnetworks, red and black, corresponding to two distinct PNGs, consisting of the same neurons. Firing of neurons *n*1 and *n*2 can trigger the whole red or black PNG: (B) If neuron *n*1 fires followed by neuron *n*2 10 ms later, then the spiking activity will start propagating along the red subnetwork, resulting in the precisely timed, i.e., *polychronous*, firing sequence of neurons *n*3,*n*4,*n*5,*n*6,*n*7, and in the short-term potentiation of the red synapses. (C) If neurons *n*2 and *n*1 fire in reverse order with the appropriate timings, activity will propagate along the black subnetwork making the same set of neurons fire but in a different order: *n*7,*n*5,*n*3,*n*6,*n*4, which temporarily strengthens the black synapses. Readout: post-synaptic neurons that receive weak connections from neurons n3, n4, and n5 with long delays and from neurons n6 and n7 with shorter delays (or, alternatively, briefly excited by the activity of the former and slowly inhibited by the latter) will fire selectively when the red *polychronous* pattern is activated, and hence could serve as an appropriate readout of the red subnetwork. A similar readout mechanism is illustrated in [53].

We propose a model in which memories are represented by extensively overlapping neuronal groups that exhibit stereotypical time-locked, but not necessarily synchronous, firing patterns called *polychronous* patterns [19] (see also [20]). In Figure 1, we use a small network to illustrate this concept: Two distinct patterns of synaptic connections (red and black connections in Figure 1A–1C) with appropriate axonal conduction delays form two distinct *polychronous* neuronal groups (PNGs). Notice that these PNGs are defined by distinct patterns of synapses, and not by the neurons *per se*, which allows the neurons to take part in multiple PNGs and enables the same set of neurons to generate distinct stereotypical time-locked spatiotemporal spike-timing patterns (see Figure 1B and 1C). PNGs arise spontaneously [19], [21] in simulated realistic cortical spiking networks shaped by spike-timing dependent plasticity [22] (STDP).

**Figure 2:**
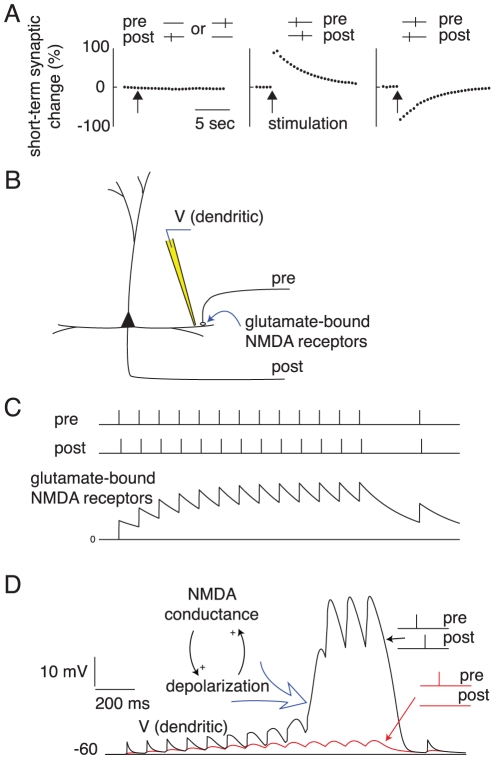
Associative short-term plasticity implemented in a form of short-term-STDP or via simulated NMDA receptors resulting in NMDA spikes. (A) The synaptic change is triggered by the classical STDP protocol at time “stimulation” (marked by arrows) but the change decays to 0 (baseline) within a few seconds. Left panel shows that firing of only pre- or post-synaptic neurons does not trigger any synaptic change. The middle panel illustrates that firing in the order pre-before-post induces short-term augmentation, as opposed to the post-before-pre (Right panel) resulting in short-term depression. (B–C) Short-term amplification of synaptic responses via simulated NMDA receptors resulting in NMDA spikes. (B) Schematic diagram showing a multi-compartmental neuron (post) receiving a synapse from a pre-synaptic neuron (pre). (C) A train of presynaptic spikes is followed by a postsynaptic response delayed by 10 ms and caused by other synaptic inputs. Each pre-synaptic spike activates postsynaptic NMDA receptors and deactivates with time constant of 250 ms. (D) Persistent pre-then-post train of action potentials flips the dendritic compartment into up-state. While in the up-state, each pre-synaptic spike results in a large-amplitude dendritic excitatory postsynaptic potential (black trace *V* (dendritic)), often called NMDA spike, that can propagate to the soma and enhance the efficacy of the synaptic transmission in eliciting somatic spike. The red trace shows the control simulation when the post-synaptic spikes are absent: No significant increase in synaptic efficacy is observed in this case. Similarly, post-before-pre patterns do not result in significant enhancement of synaptic transmission unless the timing is such that there is a residual depolarization when pre-synaptic spike arrives, or there is a residual glutamate in synaptic cleft from the previous pre-spike when post neuron fired. The voltage traces in sub-panel (D) are simulations of a passive dendritic compartment with voltage-dependent NMDA conductance.

Another distinctive feature of our theory is that synaptic efficacies are subject to associative short-term changes, that is, changes that depend on the conjunction of pre- and post-synaptic activity (see [23]–[25] for experimental findings supporting this postulation). We simulated two different mechanisms: (1) associative short-term synaptic plasticity via short-term STDP, where short-term synaptic changes — that decay to baseline within a few seconds — are induced by the classical STDP protocol (Figure 2A); and (2) the short-term amplification of synaptic responses via simulated NMDA spikes [8] at the corresponding dendritic sites (Figure 2B–2D). The latter mechanism is also pre- and postsynaptic activity dependent: Pre-synaptic spikes alone activate postsynaptic NMDA receptors, yet only generate small excitatory postsynaptic potentials (EPSPs) at the dendritic compartment (Figure 2D, red trace) because of the magnesium block of the NMDA receptors. Postsynaptic spikes, however, induce dendritic membrane potential depolarization and removal of the magnesium block. Hence, the dendritic compartment flips into up-state. While in the up-state, each presynaptic spike results in a large-amplitude response (often called an NMDA spike) that can propagate from the dendritic compartment to the soma and enhance the efficacy of synaptic transmission in eliciting somatic spikes. The short-term enhancement of synaptic efficacy is similar to that recorded in vitro [26] and in detailed simulations of Hodgkin-Huxley-type conductance-based models [27]. (See Figure 2B–2D and Methods for details.)

We found that the exact form of such short-term synaptic changes is not important for the WM functionality presented in this paper (see Results), as long as these changes selectively affect synapses according to the relative spike timing of pre- and post-synaptic neurons. For example, activation of the red PNG in Figure 1 temporarily potentiates the red synapses and not the black ones (Figure 1B and 1C). This differs from the standard short-term synaptic facilitation or augmentation used in previous WM models [7], [11], which are not associative, and hence non-selectively affect all synapses belonging to the same presynaptic neuron.

In the model presented here, PNGs get spontaneously reactivated due to stochastic synaptic noise. Short-term strengthening of the synapses of selected PNGs can bias these reactivations, i.e., increase the reactivation rate of the selected PNGs, which results in activity patterns similar to those observed *in vivo* during WM tasks [1]–[4], [13]. Additionally, even though PNGs share neurons with other PNGs, the activity of one PNG does not spread to the others. Therefore, frequent reactivation of a selected PNG does not initiate uncontrollable activity in the network. In this way, the WM mechanism presented here can work in finite networks with large memory content. This is different from previous models [11], [28]–[31] where large memory content and maintenance of several memory items can only be achieved by a drastic increase in the size of the network or the number of connections between neurons.

## Results

### The Simulated Network

**Figure 3:**
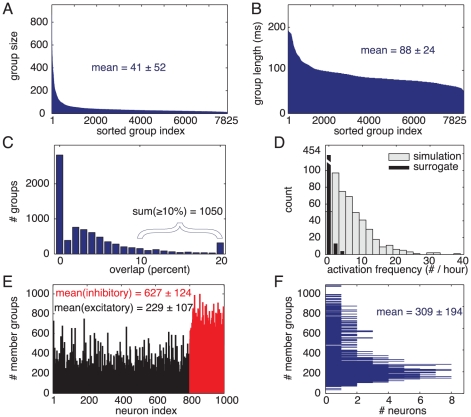
Properties of the emerging *polychronous* neuronal groups. (A) The number of emerging distinct PNGs equals 7825 for the network/simulation used (described in Methods). On average, a PNG consists of 41 neurons, (B) and their average duration is 88 milliseconds. (C) Each PNG shares at least 10 neurons, on average, with 1050 other groups. 5% of neurons of any particular group are shared with any other group in the network (not shown). (D) Distribution of frequencies of activation of PNGs in the simulated and surrogate (inverted time) spike trains. Surrogate data emphasize the statistical significance of these events. Modified with permission from [19]. (E, F) Each neuron participates in 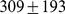 different groups.

We implemented our model in a simulated network of 1000 spiking neurons [32], where 80% of the neurons are regular spiking pyramidal neurons and 20% are GABAergic fast spiking interneurons. The probability that any pair of neurons are connected equals 0.1. Excitatory synaptic connections have a random distribution of axonal conduction delays in the [1…20] ms range [19], [33]–[35]. Excitatory synaptic efficacy is subject to both associative short-term plasticity and long-term STDP [22]. Maximum synaptic strengths are set so that three simultaneously arriving pre-synaptic spikes are needed to reliably elicit a post-synaptic spike. (The Methods section has detailed description of the network, neuron model, and synaptic plasticity.) Approximately 8000 strongly overlapping PNGs emerge spontaneously in such network (Figure 3) and we select a few to demonstrate how these mechanisms (PNG formation and associative short-term plasticity) can serve to maintain WM, and how they can account for the other related experimental findings.

### One Cue in Working Memory

**Figure 4:**
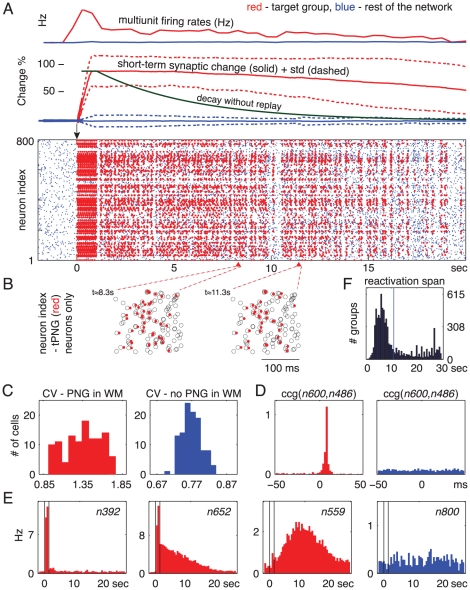
Spike timing nature of working memory - Maintenance of a *polychronous* neuronal group in working memory. (A) Bottom: Spike raster of a single trial: Blue dots, firing of all excitatory neurons in the network (inhibitory neurons not shown); Red dots, spikes of the neurons belonging to the selected target PNG (tPNG) during reactivations of the tPNG. tPNG activated in WM at 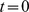 seconds (see Methods). (A) Top: Average multiunit firing rate and short-term synaptic change for tPNG (red) and for the rest of the excitatory neurons (blue). The green curve illustrates how the short-term change would decay back to baseline in the absence of neural activity after stimulation. (B) Magnified spike rasters of two partial reactivations of the tPNG neurons at two different times: Red dots, spikes of tPNG neurons; Circles, expected firings (see Methods) of all neurons in the tPNG. Only neurons belonging to the tPNG are shown. (C) CV, inter-spike interval variability histogram for tPNG neurons: Red, tPNG in WM (notice high CV values); Blue, spontaneous network activity, no PNG in WM (spike raster not shown). (D) Cross-correlograms of two neurons from the tPNG: Red, tPNG in WM; Blue, spontaneous network activity. (E) Average firing rate histogram of three representative tPNG neurons (red) while the tPNG in WM, and of a control neuron (blue) from the rest of the network. (F) Histogram of the duration of PNGs put separately in WM: time of the last reactivation (after the offset of stimulation) of each PNG versus number of PNGs with a given maximum reactivation span.

To initiate sustained neuronal activity that characterizes WM, we select (cue) a random PNG and stimulate its neurons in the sequence that characterizes the PNG's *polychronous* pattern. That is, we stimulate the intra-PNG neurons sequentially with the appropriate *polychronous* pattern 10 times during a one second interval (see e.g. Figure 4A) to temporarily increase the intra-PNG synaptic efficacy (see Methods). The red dots in the spike raster in Figure 4A indicate spikes of the selected target PNG. The initial stimulation of the target PNG resulted in short-term strengthening of the intra-PNG synapses via associative short-term plasticity, but had little effect on the other synapses in the network (Figure 4A, “short-term synaptic change” curves). Upon termination of the stimulation, the temporarily facilitated intra-PNG synapses and the noisy synaptic inputs resulted in sporadic reactivations of different segments of the target PNG, often leading to the reactivation of the rest of the *polychronous* sequence (seen as red vertical stripes in the raster in Figure 4A and magnified in Figure 4B). Each such reactivation of the target PNG triggers further strengthening of its synapses, thereby maintaining the target PNG in the active state for tens of seconds. Notably, the active maintenance of a PNG in WM does not depend on a reverberant/looping circuit, but it emerges as a result of the interplay between non-specific noise (which spontaneously triggers activation of PNGs) and short-term strengthening of the appropriate synapses (that makes subsequent reactivations of the target PNG more likely). There are frequent gaps of hundreds of milliseconds between spontaneous reactivations of the target PNG, clearly seen in Figure 4A, but occasional reactivation is necessary to maintain the PNG in WM. Without the reactivations, the initial short-term strengthening of intra-PNG synapses decays quickly (illustrated in Figure 4A, “decay without replay” curve). Figure 4F shows that almost all of the thousands of emerged PNGs, if stimulated, remained activated for more then ten seconds in WM (average 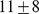 seconds).

### Precise Spike-Timing, Inter-Spike Interval Variability, and Functional Connectivity Changes during Working Memory Maintenance

**Figure 5:**
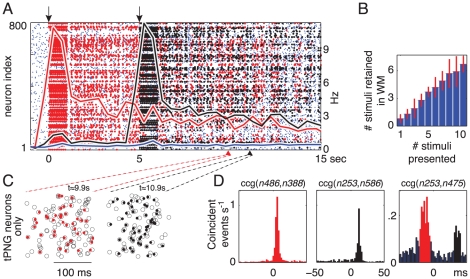
Multiple overlapping *polychronous* neuronal groups in working memory. (A) Spike raster and firing rate plots as in Figure 4. The first, red target PNG (tPNG) is activated at time 0 seconds; the second, black tPNG at time 5 seconds. The two PNGs co-exist in WM even though they share more than 25% or their neurons, which fire with different polychronous patterns. (B) Capacity tested by multiple items in WM. (C) Magnified plot of the spike rasters (red/black dots) of partial activation of the two tPNGs — red (left) and the black (right). Notation as in Figure 4B. (D) Red, left: cross-correlograms of two neurons that are part of the red but not the black PNG, when only the red PNG is in WM (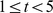 sec). Black, middle: cross-correlograms of neurons that are part of the black but not the red PNG, when only the black PNG is in WM (spike raster not shown). Right: cross-correlograms of two neurons, one from each target PNG, when both PNGs are in WM (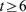 sec).

Since spontaneous reactivations of the target PNG in WM are stochastic, timing of the spiking activity of each neuron in a PNG also looks random when considered in isolation. The coefficient of variation (CV) of inter-spike intervals (ISIs), i.e., the variability of ISIs (see Methods), is higher for individual intra-PNG neurons when the PNG is in WM [36] (Figure 4C and Figure S6). This phenomenon is due to the systematically changing and non-stationary mean firing activities and mean ISIs of the intra-PNG neurons during replay (see section below). Relative intra-PNG timing at the millisecond timescale is, however, maintained during replay, as can be seen in the magnified spike rasters in Figures 4B and 5C. This is a major feature that distinguishes our approach from earlier approaches that posit synchronous [11] or totally asynchronous [7] spiking, and this feature allows our model to have a vast repertoire of overlapping PNGs, i.e., large memory content. Cross-correlograms (CCG) of simulated intra-PNG neuronal pairs also reveal the precisely timed nature of their spiking activity, as well as the context-dependent changes in functional connectivity linking these neurons: The red CCG in Figure 4D is recorded while the target PNG is in WM, and it has a peak around 5 ms, whereas the blue CCG (recorded later in a different session, when the PNG is not activated) is flat. A similar dependence of CCGs of spiking activity on the behavioral state of the network biased by sensory cues was reported in medial prefrontal neurons [37].

### Systematically Varying Persistent Firing Activity

The average multiunit firing rate of the neurons forming the target PNG following activation is around 4 Hz, much higher than that of the rest of the network, which is about 0.3 Hz (Figure 4A, “multiunit firing rate” red vs. blue solid lines). The average firing rate histograms of most intra-PNG neurons show distinct temporal profiles that repeat from trial to trial (Figure 4E and Figure S4): Some neurons only respond to the initial stimulation (Figure 4E *n*392); some have ramping or decaying firing rates (*n*652); whereas others have their peak activity seconds after the stimulus offset (*n*559). Neurons that are not part of the target PNG show uniform low firing rate activity across the whole trial (*n*800). These systematically varying, persistent temporal firing profiles are similar to those observed experimentally *in vivo* in frontal cortex during the delay period of the WM task [1], [3], [38], [39], but no previous spiking model of WM could reproduce them.

To get the results presented in Figures 4E, only an initial segment of the target PNG is activated during the selection (cueing) process (see Methods). Therefore, only the synapses forming the initial segment of the target PNG get temporarily potentiated. Hence, directly after stimulation/cuing only the neurons in the initial segment of the target PNG get more frequently reactivated as propagation of activation along the PNG dies out somewhere in the middle of the PNG without activating the neurons at the back. As frequent spontaneous reactivations persist, more and more synapses undergo short-term STDP, and more and more neurons from the end of the target PNG start to participate in the reactivations. Activities of such neurons show ramping up firing rates (Figure 4E *n*559). Conversely, neurons in the initial segment of the PNG may not participate in enough reactivations and, therefore, synapses to those neurons decay back to their baseline strength, resulting in a ramping down firing profile (*n*392 Figure 4E). In general, the slowly changing firing rates are generated by spontaneous incomplete activations within the target PNG: Neurons that are initially stimulated typically do not get reactivated or get reactivated only shortly after the target PNG stimulation and, therefore, exhibit ramping down firing profile (*n*392, *n*652); In contrast, those that join just later the wave of reactivation (Figure S4E) express ramping up (and later down) firing activity (*n*559).

### Working Memory and Timing

These stereotypical firing rate profiles may be utilized to encode time intervals [38], [40]. For example, a motor neuron circuit that needs to execute a motor action 10 seconds after a GO signal might have strong connections from neurons such as *n*559 in Figure 4E, and be inhibited by the activity of neurons such as *n*652. Moreover, a sequence of behaviors could be executed by potentiating connections from multiple subsets of the PNG to multiple motor neuron circuits (e.g., via dopamine-modulated STDP [41]). Activations of multiple representations in WM, as illustrated in Figure 5, could implement multiple timing signals and multiple sequences of actions.

### Multiple Cues in Working Memory

In a single network, multiple PNGs, i.e., multiple memories, can be loaded and maintained in WM simultaneously despite large overlap in their neuronal composition. In Figure 5A we stimulate two PNGs sequentially (out of the thousands available PNGs). The target PNGs consisted of 220 and 191 neurons each, and have 66 neurons in common. The intra-PNG neurons, however, fire with different timings relative to the other neurons within each PNG (Figures 5C and 5D). Therefore, there is little or no interference, and both PNGs are simultaneously kept in WM for many seconds. The model can hold several items in WM but eventually its performance deteriorates with increased load (note the sub-linear histogram in Figure 5B).

### Novel Stimulus - Working Memory Expands Memory Content

**Figure 6:**
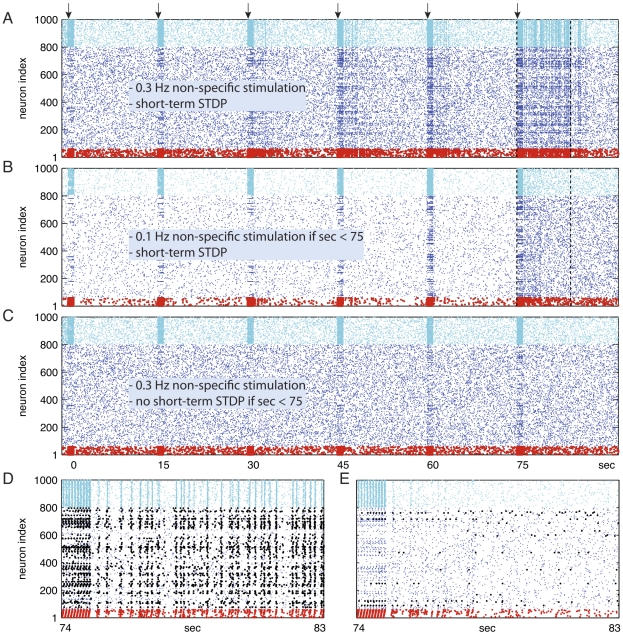
Novel cue in working memory - Formation of new *polychronous* neuronal groups. (A–C) Over 90 second long spike raster: Blue dots, spikes of excitatory neurons; Cyan dots, spikes of inhibitory neurons. Red colored dots denote the spikes of 60 randomly selected excitatory neurons that received external stimulation with a *polychronous* pattern 10 times per second every 15 seconds (arrows). The pattern used for stimulation represents the external sensory input generated by a novel cue. This pattern does not correspond to the firing pattern of any of the existing PNGs. (A) 0.3 Hz non-specific noisy minis. (B) 0.1 Hz minis when 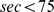. (C) Short-term STDP blocked when 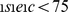. (A,B,C) Identical conditions when 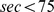. (D, E) The [74 … 83] second segment of the spike raster data of A and B are magnified in D and E, respectively. (A,D) In the presence of sufficient non-specific drive and short-term STDP, after repeated presentations a new PNG — representing the novel cue — emerges and gets frequently activated (about 4 Hz). (D) Neurons that became part of the new PNG initiated by the spiking of red neurons are marked black. The new group consists of 24 (out of 60) red and 118 black excitatory neurons. Notice that 36 of the stimulated red neurons did not become part of the newly formed PNG probably due to the lack of appropriate synaptic connections. (B,E,C) Hardly any replay in B and E, and no replay at all in C. Hampered PNG formation as WM mechanism was prevented.

To demonstrate that a novel cue can be loaded and kept in WM, we stimulated the network with a novel spike-timing pattern repeatedly every 15 seconds (Figure 6). Notice that this spiking pattern — triggered by the novel external cue — did not correspond to any of the existing PNGs' firing pattern. Each time the new pattern is presented to the network, the synapses between the stimulated neurons that fire with the appropriate order are potentiated due to long-term STDP. In addition, synapses to some other post-synaptic neurons that were firing by chance and have synaptic connections with converging conduction delays that support appropriate spike timing, are also potentiated [19]. Thus, the expansion of the network's memory content, i.e., the formation of a new PNG representing the novel cue, occurs via the interplay of long-term STDP and repeated firing of neurons with the right spatiotemporal pattern. This pattern can be triggered by stimulation (as shown in [19]), or it could result from autonomous reactivations due to WM mechanism (as shown in Figure 6A and 6D). Therefore, the WM mechanism, by facilitating the reactivations of the new PNG, facilitates the formation of the new PNG. Despite that the new PNG consists both of neurons that received (red dots in Figure 6D) and of neurons that did not receive (marked black in Figure 6D) direct stimulation during the cue presentations/learning, in order to load and keep the cue in WM it is sufficient to stimulate those neurons that were directly stimulated during learning. The reactivation rate of the new PNG, 4 Hz, is similar to those observed in Figures 4 and 5.

## Discussion

Results of our simulations are robust with respect to the mechanisms of associative short-term change of synaptic efficacies and to parameters of the model, such as short-term synaptic decay time constants (see Figures 4 and 5; and Figure S1); probability of random synaptic inputs; or choice of the target PNGs (Figure 4 and 5; see also Figures S3 and S4, where we replicate the results of Figures 4 and 5 using PNGs that were manually generated and inserted in the network (see Methods)).

The underlying currency of information in the theory presented here is the activation of a PNG. This, combined with an associative form of short-term changes of synaptic efficacies results in spontaneously emerging WM functionality: short-term synaptic changes bias the competition between PNG reactivations, and give rise to frequent spontaneous reactivations of the selected PNGs (relative to the reactivation rate of the other PNGs), which are expressed as short *polychronous* events with preserved intra-PNG spike-timings. The simulations result in a network with large memory content, and produce neural activity consistent with those observed experimentally [1], [3]. Our theory predicts that *polychronous* structures are essential for cognitive functions like WM, and such structures may be the basis for complex activity patterns observed in neocortical assemblies [42] and for memory replays involving, for example, prefrontal cortex, visual cortex, and hippocampus [43]–[45]. Additionally, this theory makes a testable prediction that changes in functional connectivity (as in Figures 4D and 5D) should be observed experimentally *in vivo* during WM tasks.

## Methods

### Neuron Model

We use a model of spiking neurons [32], [46] that was developed to satisfy two requirements: It is computational simple and efficient to implement in large-scale simulations, and it exhibits most of the types of the firing patterns recorded in animals in vitro and in vivo. We use the differential equations in the form
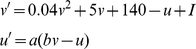
with the auxiliary after-spike resetting
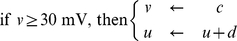
where *v* 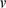 and 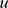 are the membrane potential and recovery variables, respectively; 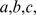 and 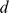 are parameters: 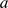, time scale of the recovery variable 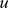; 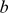, sensitivity of the recovery variable 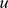 to the sub-threshold fluctuations of the membrane potential 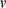; 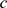, after-spike reset value of the membrane potential 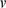 caused by the fast high-threshold 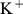 conductances; 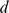, after-spike reset of the recovery variable 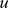 caused by slow high-threshold 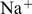 and 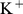 conductances.

Various choices of these parameters result in various intrinsic firing patterns, including those exhibited by the known neocortical neurons. Here 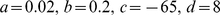 for regular spiking pyramidal neurons, and 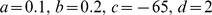 for GABAergic fast spiking interneurons. Derivation of these equations/parameters are explained in [32], [46]. 80% of the neurons in our network are regular spiking pyramidal neurons and 20% of them are GABAergic fast spiking interneurons.

### Synaptic Connections

A careful measurement of axonal conduction delays in the mammalian neocortex [33], [35] showed that these delays could be as small as 0.1 ms and as large as 44 ms, depending on the type and location of the neurons. Moreover, the propagation delay between any individual pair of neurons is precise and reproducible with a sub-millisecond precision [33], [34]. In our network (similar to the network in [19]), excitatory synaptic connections have random axonal conduction delays in the [1…20] ms range, therefore, it can be considered as a subnetwork embedded into a large part of the prefrontal cortex. All inhibitory connections are set to have 1 ms delays. The probability that any pair of neurons are connected equals 0.1.

### Synaptic Dynamics

#### Long-term dynamics

Excitatory to inhibitory and all inhibitory connections are non-plastic. Excitatory synaptic strengths change according to the STDP rule [47]. That is, the magnitude of change of synaptic strength between a pre- and a postsynaptic neuron depends on the timing of spikes: The synapse gets potentiated if the presynaptic spike arrives at the postsynaptic neuron before the postsynaptic neuron fires; Whereas, the synapse gets depressed if the presynaptic spike arrives at the postsynaptic neuron after that fired. Thus, what matters is not the timing of spiking *per se* but the exact timing of the arrival of presynaptic spikes to postsynaptic targets. Formally, the magnitude of change for potentiation equals 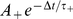; and for synaptic depression is 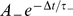, where 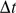 is the inter-spike interval between the arrival of the presynaptic spike and the postsynaptic spike, 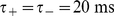, 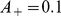, and 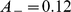. The synaptic strengths are bound within the interval [0…8] mV, which implies that the simultaneous arrival of at lease three pre-synaptic spikes are needed to reliably elicit a post-synaptic response. About 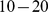 optimal pre-then-post spike pairs are needed to increase the synaptic strength of a weak synapse to the maximum value.

#### Short-term dynamics

The efficacy of synaptic transmission for synapses connecting excitatory neurons are also scaled up or down, relative to a baseline, on a short timescale. We implement these short term dynamics in two form: short-term STDP and NMDA spikes.

*Short-term STDP*: Without short-term changes, input to neuron 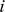 at time 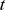, 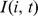, equals 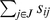, where 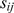 is synaptic weight for the synapse between neuron 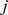 and 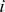; and 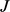 is the set of presynaptic neurons whose spike arrived at neuron 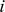 at time 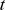.

With short-term STDP the input changes to
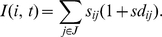
That is, the effect of a presynaptic spike is scaled up or down by the factor sd, where this 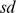 variable is different for each synapse; follows the classical STDP rule; and in the absence of synaptic activity it decays back to 0 with a time constant 5 seconds. Therefore, 1) in the absence of synaptic activity the synaptic efficacy does not change; 2) pre-then-post spikes temporarily increase the synaptic efficacy; and 3) post-then-pre spikes temporarily decrease the synaptic efficacy. About 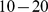 optimal pre-then-post spike pairs are needed to gain a maximum of 100% temporary increase relative to the baseline.

*NMDA spikes*: The voltage traces in Figure 2C and 2D are simulations of a passive dendritic compartment with voltage-dependent NMDA conductance. Parameters (see [46] for detailed description of conductance based models): 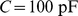, 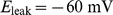, 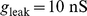, 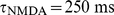, 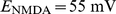; The voltage dependence of NMDA conductance is described by the nonlinear function 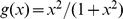 if 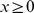 and 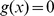 if 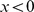, where 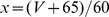 and 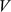 is the dendritic membrane potential. The NMDA current is 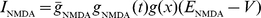, where 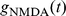 is the time-dependent activation of NMDA channels due to synaptic input, and 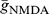 is the maximal conductance.

We select 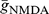 so that the NMDA to AMPA current ratio is 9 to 1 at the fully depolarized postsynaptic potentials, resulting in 10-fold increase in the effectiveness of the synaptic transmission and in the hysteresis of NMDA current: Once 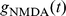 is above certain threshold value 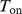 and there is a somatic spike at the postsynaptic compartment, the postsynaptic membrane potential depolarizes enough to turn on the NMDA current. The current remains on via positive feedback loop (Figure 2D), and the postsynaptic potential remains depolarized, as long as 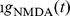 is above certain lower threshold 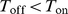. The current turns off when 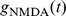 falls below the lower threshold, i.e., the positive feedback is no longer capable to maintain the depolarization needed to remove the magnesium block of NMDA channels.

We assume here that each synapse has its own postsynaptic compartment with its own 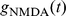, which is independent from its neighbors. This conductance is increased by each arriving spike and exponentially decays with the time constant of 250 ms. To speed-up simulations of the network of 1000 neurons and to avoid having 100,000 compartments, we model the NMDA synapses via a hysteresis function: The synaptic efficacy is 10-fold tronger when 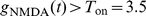 and there is a post-synaptic spike, and it returns to normal values when 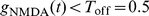. This results in an associative short-term plasticity, as the strength of synaptic transmission between two neurons can be transiently increased if the post-synaptic neuron fires persistently after the pre-synaptic one.

Figure S1 demonstrates the NMDA spikes based WM mechanism. For the figures in the main text short-term STDP was used. Long-term STDP was used for the PNG formation, but for demonstration purposes in Figure 4 and 5 the long-term plasticity is blocked. In Figure 6 long-term and short-term STDP work in parallel.

### Finding *Polychronous* Neuronal Groups

After running the simulation for five hours, providing only random synaptic input to the network, we analyzed the evolved network data — synaptic connections, axonal conductance delays, and synaptic strengths — using the methods described in [19] and found a total of 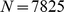 spontaneously generated, strongly overlapping distinct PNGs; See Figure 3 for details on the emerging PNGs. We used these spontaneously emerging PNGs for the results shown in Figures 4 and 5.

Embedded in the noisy spike train are occasional precise spiking patterns corresponding to spontaneous reactivations of PNGs [19]. Since each such PNG has a distinct pattern of *polychronous* spiking activity, we use the pattern as a template to find the reactivation of the PNG in the spike train. A PNG is said to be activated when more than 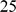 percent of its neurons fire according to the prescribed *polychronous* pattern with 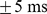 jitter.

### Stimulating a PNG

To select a specific PNG in WM, i.e, to temporarily increase the intra-PNG synaptic efficacy, we transiently stimulate its neurons sequentially with the appropriate spatiotemporal spike-timing pattern [48]–[50]. What enters WM is possibly gated by attention. To avoid modeling attentional mechanisms, we provide two different gating implementations:

1. Stimulate the intra-PNG neurons sequentially with the appropriate *polychronous* pattern 10 times during a one second interval (as seen in Figures 4A and 5A) to temporarily increase the intra-PNG synaptic efficacy. This simulates the arrival of visually evoked volleys of spikes due to several micro-saccades per second [50].
2. Stimulate the intra-PNG neurons sequentially with the appropriate *polychronous* pattern but only one to three times in the presence of elevated level of a simulated neuromodulator, e.g., dopamine, that increases the synaptic plasticity rate (as in Figure S2). This stimulation mechanism results in a 5-fold faster rate of change of synaptic plasticity [51]. Dopaminergic regulation of prefrontal cortex activity is essential for cognitive functions such as working memory [52]. Elevated neuromodulator level in this implementation increases the level of sensitivity of WM to the current stimulus.

We also performed stochastic stimulations (for both types of stimulations) where the firing response probability of individual neurons to external stimulation was smaller then 1 and found the qualitative behavior of the network to be similar. For example, the response probability for the target neurons in Figure S2 is 0.8.

For the results presented in Figures 4E (and 6), and Figures S3 and S4, not all the neurons of the target PNG were stimulated (with the appropriate *polychronous* pattern) but only the initial segment of the target PNGs (80 percent in Figure 4E; 10 percent in Figures S3 and S4). The rest of the target neurons (i.e., neurons that were not stimulated but are part of the target PNG) systematically joined the reactivation process. (For detailed description, see the figure legends for Figures S3 and S4.)

### Inserted *Polychronous* Neuronal Groups

For the results presented in Figures S3 and S4 we inserted additional synapses in the randomly connected network in order to form 100 new PNGs. Activity of each such PNG lasted for 200 milliseconds and it consisted of 40 neurons. Each intra-PNG neuron has at least three converging synapses from other pre-synaptic intra-PNG neurons (except for the first three neurons in the PNG).

### Non-Specific Input to the Network

Throughout the whole simulation the network is stimulated with stochastic miniature synaptic potentials (called “minis”), and it exhibits asynchronous noisy spiking activity. The average background multiunit firing rate is around 0.3 Hz for the simulations presented in the article. Qualitative behavior of the network is similar to a wide range of noisy background firing activity, which, however, cannot be too small, as some background activity is necessary to initiate spontaneous PNG reactivations (see Figure 6 and Figure S5), or too high, as that would interfere with neural activity propagation within the PNG.

Spontaneously emerging PNGs in the simple network we used tend to be prone to noise. This means that the initiated activity in the PNG is less likely to propagate along the whole PNG in the presence of high background noise (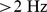 for excitatory neurons). This is because neurons that should respond (fire) to presynaptic activity and pass that activity to postsynaptic intra-PNG neurons are likely to be inhibited or be in their refractory period if there is too much background activity present in the network.

Manually inserted PNGs can be engineered to have redundant connections, i.e, postsynaptic neurons have more presynaptic connections (from multiple presynaptic neurons) than minimally required to fire these postsynaptic neurons. This redundancy can make these PNGs much more robust to noise: the inability of a presynaptic neuron to fire (e.g. due to inhibition) is less likely to prevent the propagation of activity in the PNG, as there are likely other presynaptic intra-PNG neurons firing and passing the activity to the same postsynaptic target.

### CV - Variability of Inter-Spike Intervals

The first 20 seconds after stimulus presentation offset of the spike trains of the target PNG were used for inter-spike interval (ISI) analysis presented in Figure 4C and Figure S6, red histograms. The data was collected over multiple trials. The coefficient of variation (CV) measures the variation in the neurons' ISIs: 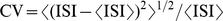, i.e., CV equals the standard deviation of ISIs divided by the mean ISI. 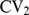, a local measure for coefficient of variation, used for Figure S6 is less biased by non-stationary ISIs. 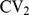 is computed by comparing each ISI (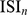) to the subsequent ISI (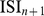) to evaluate the degree of variability of ISIs in a local manner: 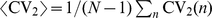, where 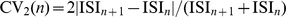. These measures are identical to those used in [36].

## Supporting Information

Figure S1 Maintenance of a polychronous neuronal group in working memory with short-term amplification of synaptic responses via NMDA spikes - One trial. Neurons of the target PNG (to be loaded into WM) are stimulated with the appropriate spike-timing pattern repeated 10 times, starting at t = 0 seconds - similar to the mechanism used in Figures 4 and 5 of main text. Solid lines: average multiunit firing rate of the target group (red) and that of the rest of the excitatory neurons (blue). Blue dots, spikes of excitatory neurons; Cyan dots, inhibitory neurons; Red dots, spikes of the neurons belonging to the target group during [partial] reactivations of the target group, that is, when more than 25 percent of its neurons fire with the expected (±5 ms) spatiotemporal pattern. Dark green line, time course of the short-term synaptic decay without spontaneous replay of the target group; time constant is 250 milliseconds.
(0.81 MB TIF)

Figure S2 Increased plasticity rate modulated by elevated level of a simulated neuromodulator. (A) Spike raster and firing rate plots during a single WM task/trial. Solid lines: average multiunit firing rate of the target group (red) and that of the rest of the excitatory neurons (blue). Blue dots, spikes of excitatory neurons; Cyan dots, inhibitory neurons; Red dots, spikes of the neurons belonging to the target PNG during [partial] reactivations of the target group, that is, when more than 25 percent of its neurons fire with the expected (±5 ms) spatiotemporal pattern. The target PNG was stimulated at 0 second and at 5 seconds (shading). The brown shaded area starting a little before 5 seconds (better seen in subplots B and C) denotes an elevated simulated neuromodulator level, which results in 5 times faster plasticity change in the network. Therefore, fewer PNG stimulation (three in this example) is enough to temporary increase the intra-PNG synaptic efficacy and trigger WM functionality. (B) Data and notation as in A but only neurons of the target groups in the [5 … 10] second interval are shown. Data in C is identical to B but the plotting of the neurons is reordered so their polychronous firing is clearly visible as tilted lines.
(1.15 MB TIF)

Figure S3 Maintenance of multiple representations in working memory in a network with 100 embedded PNGs. The spike raster shows only excitatory neurons participating in neuronal groups A_13_, A_92_, A_1_, and A_2_. Activation of each such neuronal group, involving more than 25 percent of its neurons is marked by spikes of different color. Insets show raster plots corresponding to partial activation of various neuronal groups. Circles show where the spikes are expected, black dots show the actual spikes. The network exhibits spontaneous activity except at 0 second (stimulation of the first ten neurons belonging to A_1_) and 10 seconds (stimulation of the first ten neurons belonging to A_2_). If a few neurons forming the i^th^ PNG, A_i_, fire with the appropriate spike-timing, the rest of the neuronal group responds with the corresponding polychronous firing pattern. For example, the left two inserts show spontaneous activation of A_13_ and A_92_. To select a PNG to be held in working memory we activate it by an appropriate sensory input. For example, at time 0 seconds we stimulated the first 10 neurons of the sequence A_1_ with the appropriate timing 10 times per second during the interval of 1 second. (Notice that the first four stimulations are not colored as less then 25 percent of the A_1_ was activated.) This stimulation resulted in short-term strengthening of the synaptic connections forming the initial segment of A_1_ via short-term STDP, but had little effect on the other synapses. Upon termination of the simulated applied input, the strengthened intra-group connectivity resulted in the spontaneous reactivation of the initial segment of A_1_ with the precise timing of spikes (3^rd^ inset), leading often to the activation of the rest of the sequence (marked by red dots). Each such spontaneous reactivation of A_1_ results in further strengthening of the synaptic connectivity forming A_1_, thereby maintaining A_1_ in the “active” state for tens of seconds. Notice that such an active maintenance is accomplished without any recurrent excitation. Even though each neuron in A_1_ fires with a precise timing with respect to the other neurons in the PNG, the activity of the neuron looks random. To illustrate maintenance of multiple memory representations in working memory, we stimulate the initial segment of group A_2_ with a 10 Hz 1 sec long specific excitatory drive. Even though the neuronal groups A_1_ and A_2_ partially overlap, the neurons fire with different timings relative to the other neurons within each group, so there is little or no interference, and both representations are kept in working memory for many seconds.
(0.81 MB TIF)

Figure S4 Systematically changing persistent firing rates during working memory tasks. Spike rasters and mean (over several trials) firing rates of neurons at the beginning (A), middle (B) and the end (C) of the polychronous sequence forming the neuronal group A_1_ (see Figure S3), and a control neuron (D) not belonging to the PNG. Arrow marks the trigger stimulus. The firing rates of these neurons have stereotypical profiles that are reproducible from trial to trial (as are often those observed experimentally. Sensory stimuli were needed to activate only the initial part of the corresponding PNG (network noise prevents full activation of the sequence), resulting in high firing rate in A, but low initial rates in B and C. Subsequent spontaneous reactivations resulted in stronger synapses and in longer sequences (insets in Figure S3) leading to the steady increase in the firing rates (B and C lower panel). Often, reactivation starts in the middle of the sequence, thereby strengthening synapses downstream but not affecting synapses upstream of the sequence. Eventually, the synaptic connections forming the initial segment become weaker and that part of the neuronal group stops reactivating, resulting in the decline in the firing rate as seen in A and then in B. (E) Neurons in A_1_ are sorted according to their relative spike-timing within the polychronous sequence and show a single trial spike raster. A slowly traveling wave (moving hot spot) of increased firing rates is generated by spontaneous incomplete activations within A_1_. This wave could provide a timing signal to a separate brain region to execute a behavior or a sequence of behaviors locked to the onset of the trigger stimulus. For example, a motor neuron circuit that needs to execute a motor action 10 seconds after the trigger should have strong connections from neurons 20 through 30 from the neuronal group, but be inhibited by the activity of neurons 1 through 20. A sequence of behaviors could be executed by potentiating connections from multiple subsets of the neuronal group to multiple motor-neuron circuits (e.g., via dopamine-modulated STDP [Izhikevich E.M., 2007, Solving the distal reward problem through linkage of stdp and dopamine signaling. Cereb Cortex 17: 2443–52.]). Similarly, activations of multiple representations in short-term memory, as in Figure S3 (sec>15) and Figure 4 (main text), would implement multiple clocks and multiple sequences of actions.
(0.57 MB TIF)

Figure S5 Interrupting the replay of PNGs maintained in WM. Working memory functionality in our model emerges via the interplay between spontaneous synaptic input (minis) and short-term synaptic plasticity. Blocking the minis or diminishing the effect of short-term plasticity can interrupt the replay process, which provides a mechanism to remove an item from WM. Spike raster and firing rate plots as in Figures 4 and 5 of main text. At time 5 seconds, as an effect of change in a simulated neuromodulator level, the short-term plasticity rate fades and, therefore, the reactivation of the target PNG stops and the strength of synapses of the target PNG decay to their baseline.
(0.20 MB TIF)

Figure S6 Global versus local measures of CV. Upper row: global CV (see Methods in main text for details); Results similar to those in Figure 3C in main text. Lower row: CV_2_, a local measure of CV (see Methods). The firing profile and the mean ISI of intra-PNG changes systematically when the PNG is in WM (Figure 4E in main text and Figure S4). Therefore, the ISIs during the replay period are non-stationary, which results in high CV values (upper left histogram).
(0.13 MB TIF)
