# Supplementary figures and images for: Spike-Timing Theory of Working Memory
Source: PLoS Comput Biol. 2010 Aug 19;6(8):e1000879. doi: 10.1371/journal.pcbi.1000879 (PMC2924241; doi:10.1371/journal.pcbi.1000879)

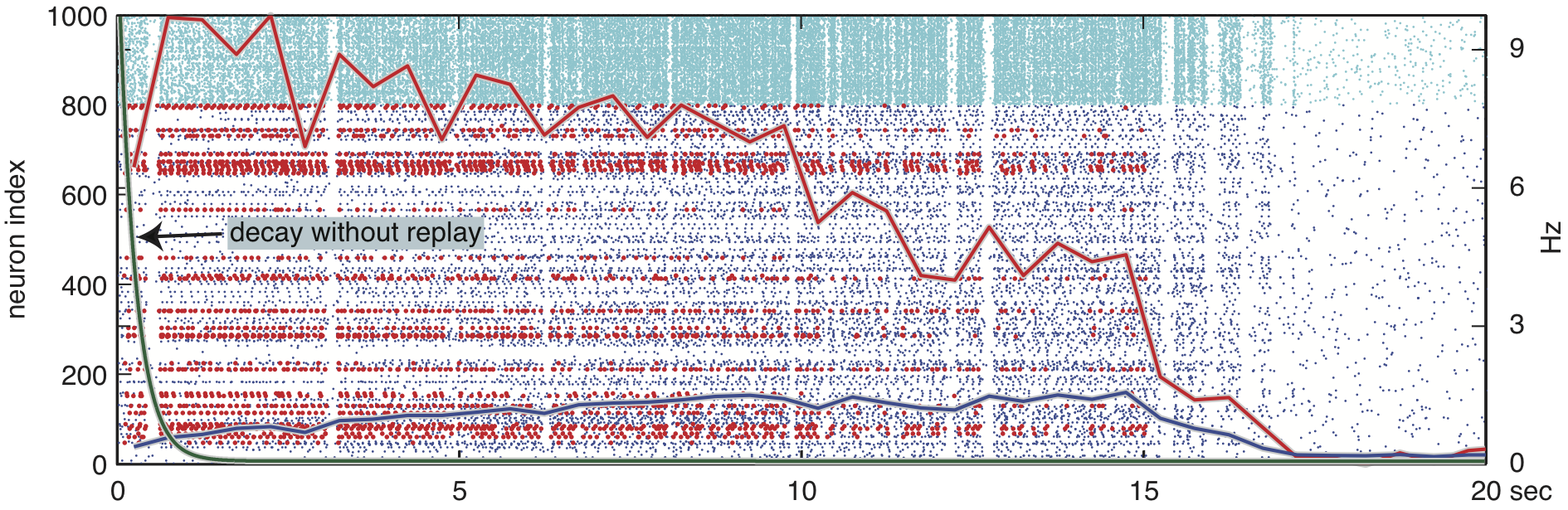

Supplement: Figure S1 — Maintenance of a polychronous neuronal group in working memory with short-term amplification of synaptic responses via NMDA spikes - One trial. Neurons of the target PNG (to be loaded into WM) are stimulated with the appropriate spike-timing pattern repeated 10 times, starting at t = 0 seconds - similar to the mechanism used in Figures 4 and 5 of main text. Solid lines: average multiunit firing rate of the target group (red) and that of the rest of the excitatory neurons (blue). Blue dots, spikes of excitatory neurons; Cyan dots, inhibitory neurons; Red dots, spikes of the neurons belonging to the target group during [partial] reactivations of the target group, that is, when more than 25 percent of its neurons fire with the expected (±5 ms) spatiotemporal pattern. Dark green line, time course of the short-term synaptic decay without spontaneous replay of the target group; time constant is 250 milliseconds. (0.81 MB TIF) [file pcbi.1000879.s001.tif]

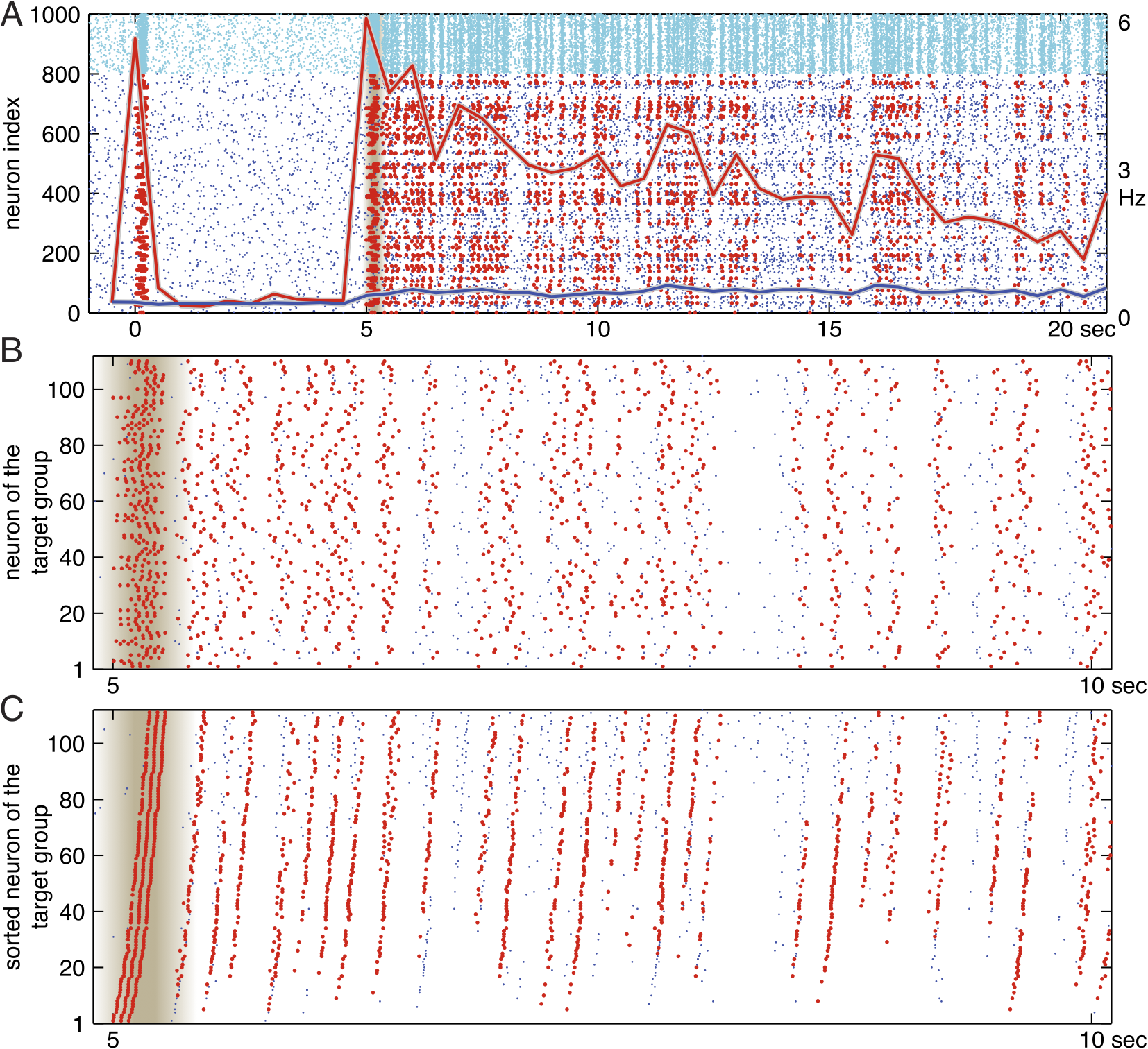

Supplement: Figure S2 — Increased plasticity rate modulated by elevated level of a simulated neuromodulator. (A) Spike raster and firing rate plots during a single WM task/trial. Solid lines: average multiunit firing rate of the target group (red) and that of the rest of the excitatory neurons (blue). Blue dots, spikes of excitatory neurons; Cyan dots, inhibitory neurons; Red dots, spikes of the neurons belonging to the target PNG during [partial] reactivations of the target group, that is, when more than 25 percent of its neurons fire with the expected (±5 ms) spatiotemporal pattern. The target PNG was stimulated at 0 second and at 5 seconds (shading). The brown shaded area starting a little before 5 seconds (better seen in subplots B and C) denotes an elevated simulated neuromodulator level, which results in 5 times faster plasticity change in the network. Therefore, fewer PNG stimulation (three in this example) is enough to temporary increase the intra-PNG synaptic efficacy and trigger WM functionality. (B) Data and notation as in A but only neurons of the target groups in the [5 … 10] second interval are shown. Data in C is identical to B but the plotting of the neurons is reordered so their polychronous firing is clearly visible as tilted lines. (1.15 MB TIF) [file pcbi.1000879.s002.tif]

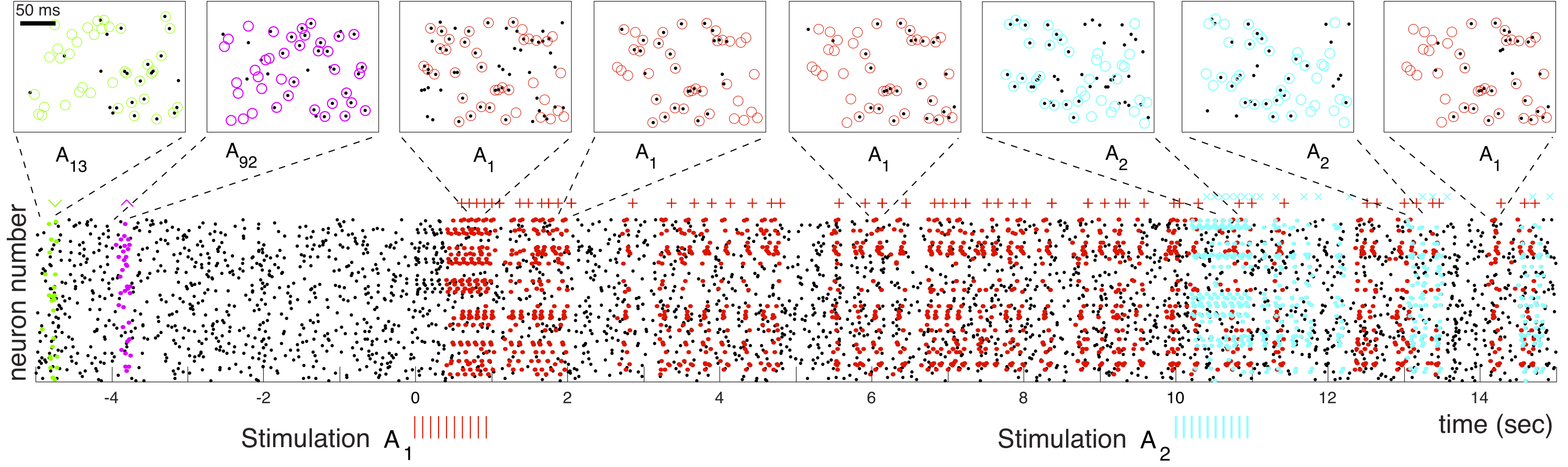

Supplement: Figure S3 — Maintenance of multiple representations in working memory in a network with 100 embedded PNGs. The spike raster shows only excitatory neurons participating in neuronal groups A13, A92, A1, and A2. Activation of each such neuronal group, involving more than 25 percent of its neurons is marked by spikes of different color. Insets show raster plots corresponding to partial activation of various neuronal groups. Circles show where the spikes are expected, black dots show the actual spikes. The network exhibits spontaneous activity except at 0 second (stimulation of the first ten neurons belonging to A1) and 10 seconds (stimulation of the first ten neurons belonging to A2). If a few neurons forming the ith PNG, Ai, fire with the appropriate spike-timing, the rest of the neuronal group responds with the corresponding polychronous firing pattern. For example, the left two inserts show spontaneous activation of A13 and A92. To select a PNG to be held in working memory we activate it by an appropriate sensory input. For example, at time 0 seconds we stimulated the first 10 neurons of the sequence A1 with the appropriate timing 10 times per second during the interval of 1 second. (Notice that the first four stimulations are not colored as less then 25 percent of the A1 was activated.) This stimulation resulted in short-term strengthening of the synaptic connections forming the initial segment of A1 via short-term STDP, but had little effect on the other synapses. Upon termination of the simulated applied input, the strengthened intra-group connectivity resulted in the spontaneous reactivation of the initial segment of A1 with the precise timing of spikes (3rd inset), leading often to the activation of the rest of the sequence (marked by red dots). Each such spontaneous reactivation of A1 results in further strengthening of the synaptic connectivity forming A1, thereby maintaining A1 in the “active” state for tens of seconds. Notice that such an active maintenance is accomplis [file pcbi.1000879.s003.tif]

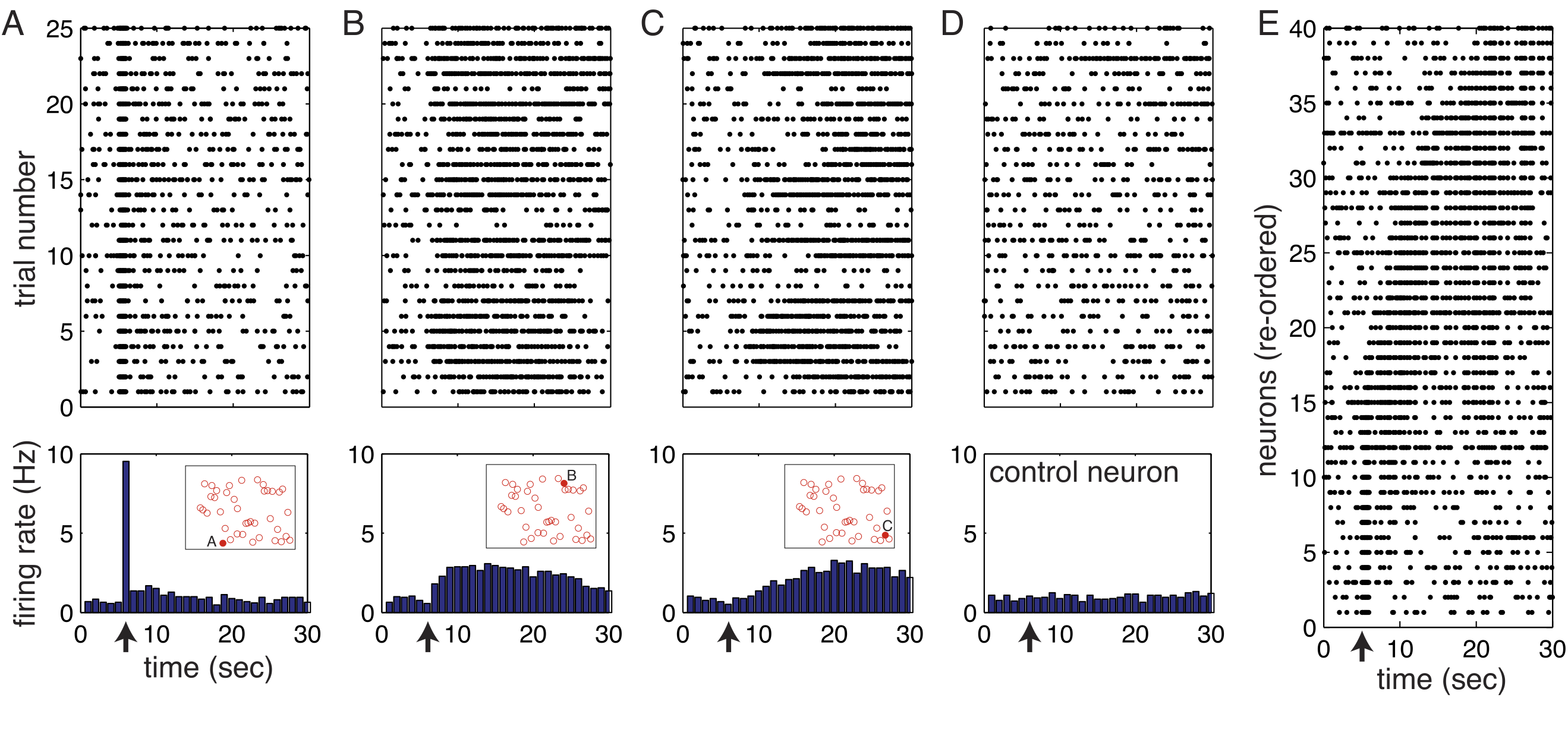

Supplement: Figure S4 — Systematically changing persistent firing rates during working memory tasks. Spike rasters and mean (over several trials) firing rates of neurons at the beginning (A), middle (B) and the end (C) of the polychronous sequence forming the neuronal group A1 (see Figure S3), and a control neuron (D) not belonging to the PNG. Arrow marks the trigger stimulus. The firing rates of these neurons have stereotypical profiles that are reproducible from trial to trial (as are often those observed experimentally. Sensory stimuli were needed to activate only the initial part of the corresponding PNG (network noise prevents full activation of the sequence), resulting in high firing rate in A, but low initial rates in B and C. Subsequent spontaneous reactivations resulted in stronger synapses and in longer sequences (insets in Figure S3) leading to the steady increase in the firing rates (B and C lower panel). Often, reactivation starts in the middle of the sequence, thereby strengthening synapses downstream but not affecting synapses upstream of the sequence. Eventually, the synaptic connections forming the initial segment become weaker and that part of the neuronal group stops reactivating, resulting in the decline in the firing rate as seen in A and then in B. (E) Neurons in A1 are sorted according to their relative spike-timing within the polychronous sequence and show a single trial spike raster. A slowly traveling wave (moving hot spot) of increased firing rates is generated by spontaneous incomplete activations within A1. This wave could provide a timing signal to a separate brain region to execute a behavior or a sequence of behaviors locked to the onset of the trigger stimulus. For example, a motor neuron circuit that needs to execute a motor action 10 seconds after the trigger should have strong connections from neurons 20 through 30 from the neuronal group, but be inhibited by the activity of neurons 1 through 20. A sequence of behaviors could be executed by potentiating [file pcbi.1000879.s004.tif]

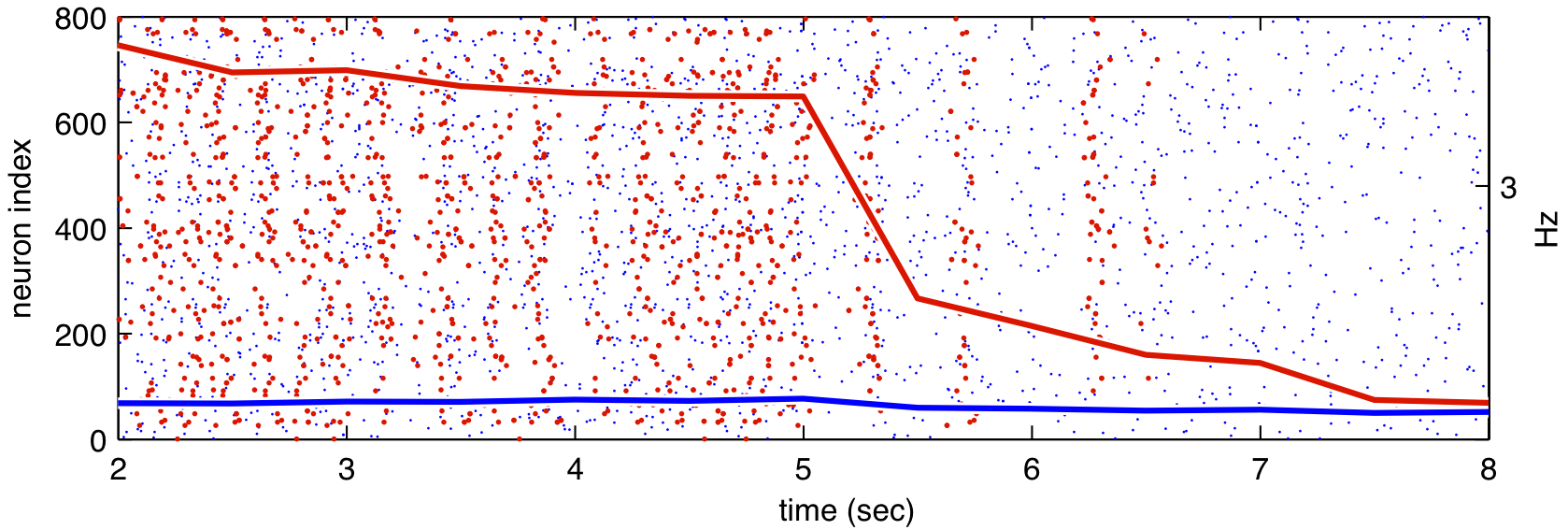

Supplement: Figure S5 — Interrupting the replay of PNGs maintained in WM. Working memory functionality in our model emerges via the interplay between spontaneous synaptic input (minis) and short-term synaptic plasticity. Blocking the minis or diminishing the effect of short-term plasticity can interrupt the replay process, which provides a mechanism to remove an item from WM. Spike raster and firing rate plots as in Figures 4 and 5 of main text. At time 5 seconds, as an effect of change in a simulated neuromodulator level, the short-term plasticity rate fades and, therefore, the reactivation of the target PNG stops and the strength of synapses of the target PNG decay to their baseline. (0.20 MB TIF) [file pcbi.1000879.s005.tif]

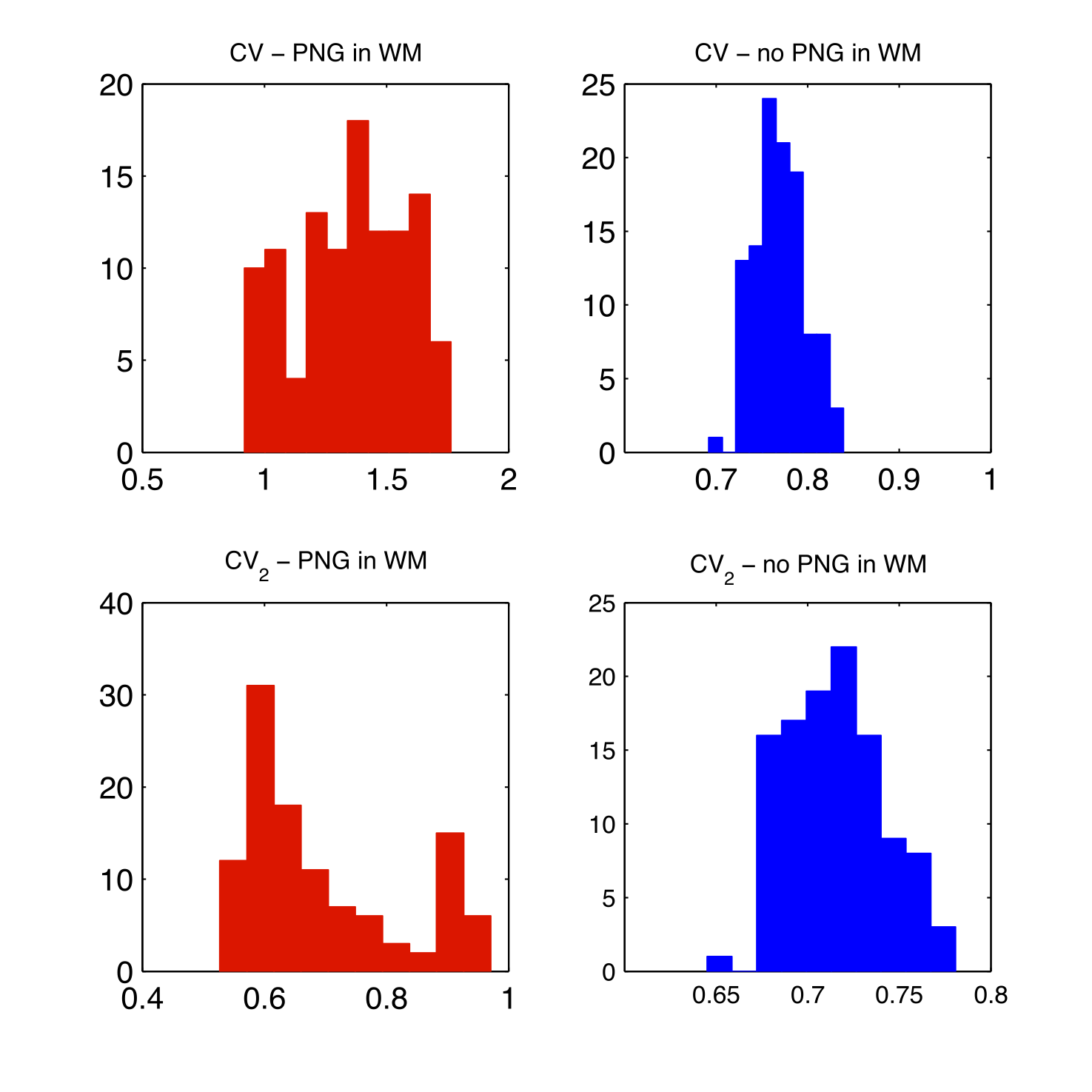

Supplement: Figure S6 — Global versus local measures of CV. Upper row: global CV (see Methods in main text for details); Results similar to those in Figure 3C in main text. Lower row: CV2, a local measure of CV (see Methods). The firing profile and the mean ISI of intra-PNG changes systematically when the PNG is in WM (Figure 4E in main text and Figure S4). Therefore, the ISIs during the replay period are non-stationary, which results in high CV values (upper left histogram). (0.13 MB TIF) [file pcbi.1000879.s006.tif]
